# Renal Physiological Adaptation to High Altitude: A Systematic Review

**DOI:** 10.3389/fphys.2020.00756

**Published:** 2020-07-16

**Authors:** Lisa M. Palubiski, Ken D. O'Halloran, Julie O'Neill

**Affiliations:** Department of Physiology, School of Medicine, College of Medicine & Health, University College Cork, Cork, Ireland

**Keywords:** tissue hypoxia, renal adaptation, hemodynamic parameters, electrolyte balance, oxygen tension

## Abstract

**Background:** Under normal physiological conditions, renal tissue oxygen is tightly regulated. At high altitude, a physiological challenge is imposed by the decrease in atmospheric oxygen. At the level of the kidney, the physiological adaptation to high altitude is poorly understood, which might relate to different integrated responses to hypoxia over different time domains of exposure. Thus, this systematic review sought to examine the renal physiological adaptation to high altitude in the context of the magnitude and duration of exposure to high altitude in the healthy kidney model.

**Methods:** To conduct the review, three electronic databases were examined: OVID, PubMed, and Scopus. Search terms included: Altitude, renal, and kidney. The broad, but comprehensive search, retrieved 1,057 articles published between 1997 and April 2020. Fourteen studies were included in the review.

**Results:** The inconsistent effect of high altitude on renal hemodynamic parameters (glomerular filtration rate, renal blood flow, and renal plasma flow), electrolyte balance, and renal tissue oxygen is difficult to interpret; however, the data suggest that the nature and extent of renal physiological adaptation at high altitude appears to be related to the magnitude and duration of the exposure.

**Conclusion:** It is clear that renal physiological adaptation to high altitude is a complex process that is not yet fully understood. Further research is needed to better understand the renal physiological adaptation to hypoxia and how renal oxygen homeostasis and metabolism is defended during exposure to high altitude and affected as a long-term consequence of renal adaptation at high altitude.

## Introduction

Tissue hypoxia can present either due to a decrease in oxygen delivery and/or as a result of an increase in oxygen consumption (Marshall et al., 1994; Patinha et al., 2017). In the context of the kidney, renal tissue oxygen tension is defined as the partial pressure of oxygen in the renal cortex and medulla. Renal tissue hypoxia is characterized by a decrease in the partial pressure of oxygen in the tissue. The complex vascular architecture of the kidney coupled with the substantial oxygen demand (owed to solute reabsorption) could predispose the kidney to hypoxia-induced injury (Fu et al., 2016). This is particularly true in the renal medulla where only 10% of the total renal blood flow is delivered, but the site where some 30% of filtered sodium chloride is reabsorbed by an energy-dependent mechanism (Hansell et al., 2013).

The vulnerability of the kidney tissue to hypoxic insult is mitigated by the robust regulatory mechanism aimed at preserving intra-renal oxygen homeostasis. Under normal physiological conditions, renal tissue oxygen tension is governed by the carefully orchestrated balance between oxygen supply via renal blood flow and demand via renal oxygen consumption. However, the relative distribution of the total renal blood flow between the cortex and the medulla does vary, which could potentially alter the regional delivery of oxygen (Eckardt et al., 2005). Thus, an alteration in the global delivery of oxygen via the renal blood flow might not be reflected at the regional level.

Glomerular filtration rate is a vital determinant of renal oxygen consumption, through its impact upon the proximal reabsorption of sodium. A quasi-linear relationship exists between sodium transport and renal oxygen consumption (Swartz et al., 1977; McTigue et al., 1983). This suggests that renal oxygen consumption could be divided into an active and basal component (Thurau, 1961; Evans et al., 2014). Approximately 80% of total renal oxygen consumption is accounted for by the active reabsorption of sodium along the nephron, while the remaining 20% is related to basal metabolism. Basal metabolism is owed to transport independent cellular processes, such as mitochondria proton leak and gluconeogenesis (Thurau, 1961; Evans et al., 2014).

More recently, it has become apparent that a sustained reduction in renal tissue oxygen tension is evident in both the diabetic and hypertensive model (Hansell et al., 2013; O'Neill et al., 2015, 2019; Franzen et al., 2016) and could potentially play an important role in the development of, or progression to, chronic kidney disease. Indeed, a recent experimental finding revealed a sustained decrease in renal tissue oxygen tension which preceded proteinuria in a diabetic animal model (Franzen et al., 2016). Furthermore, renal tissue hypoxia and nephropathy were evident in a normal rat model treated chronically with the potent mitochondrial uncoupler, dinitrophenol (Friederich-Persson et al., 2013). Both provided important evidence to show the development of renal tissue hypoxia and injury in an animal model not confounded by oxidative stress or overt hyperglycemia.

High altitude is characterized by the sustained decrease in atmospheric oxygen, with the decrease in oxygen a function of the degree of elevation (Bigham and Lee, 2014). Given the physiological challenge imposed by high altitude, renal adaptation could become overloaded leading to renal tissue injury. This led to the concern that high altitude could hasten progression to end stage renal disease in those with chronic kidney disease (CKD), even though limited available data has suggested that those with CKD can tolerate short-term exposure to modest altitude (Luks et al., 2008). However, a better understanding of the renal adaptation to the decrease in oxygen availability in the healthy model is needed before research can begin to discern the risk of high altitude in those with CKD.

At exposure to high altitude, there is a well-coordinated change in the respiratory system, cardiovascular system, and renal system each designed to improve tissue oxygenation (Goldfarb-Rumyantzev and Alper, 2014). Whereas, hypoxic responsiveness of the ventilatory system has received a lot of attention and is generally well-understood, the hypoxia-induced change in renal function (hemodynamic regulation and electrolyte balance) is much less understood. It has been established that the kidney plays a central role in the preservation/maintenance of whole body tissue oxygen perfusion. During the acute phase of the adaptatory response, the kidney indirectly increases the oxygen-carrying capacity of the blood by promoting the excretion of salt and water, resulting in hemo-concentration and thereby an increase in hematocrit. A persistent decline in cortico-medullary oxygen tension drives the chronic portion of the response and stimulates the expression and synthesis of erythropoietin (EPO) by peritubular fibroblast cells (Donnelly, 2001). Importantly, hypoxia-induced EPO expression and synthesis involves complex molecular mechanisms that are dependent upon hypoxia-inducible factor (HIF)-2 (Haase, 2010).

High altitude is a commonly used model to examine renal tissue hypoxia under normal physiological conditions and may inform disease models, as well as reveal the renal contributions and limitations to high altitude acclimatization. Thus, the current review sought to examine the change in renal physiological function as a result of exposure to high altitude. The objective of the review was to determine the effect of duration and magnitude of exposure to high altitude-induced hypoxia on renal physiological function (hemodynamic regulation and electrolyte balance) in the context of renal oxygenation in the healthy animal and human model. The second objective was to identify the gaps in the literature and inform future research directions to enhance the overall understanding of the effect of renal tissue hypoxia on both the healthy and disease kidney model.

## Methods

**Figure 1:**
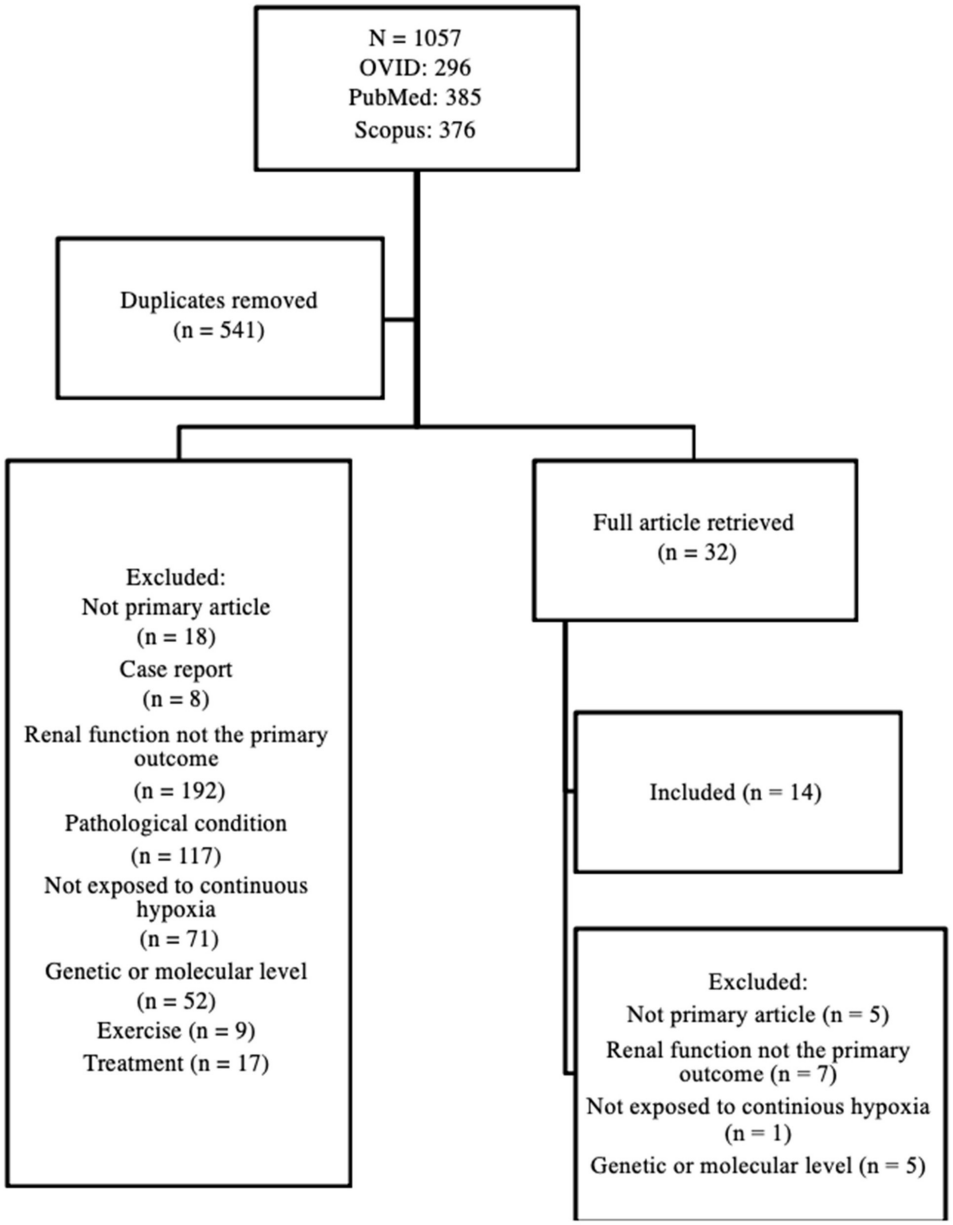
Article selection process.

A systematic review of the literature was conducted and included primary research articles that examined renal adaptation to acute and chronic high altitude-related conditions. To conduct the review, three electronic databases were examined: OVID, PubMed, and Scopus. Search terms included: Altitude, renal, and kidney. The broad, but comprehensive search retrieved 1,057 articles published between 1997 and April 2020 without language restriction. In addition to the above, one article was identified from the reference list of a selected article (via footnote chasing). The title and abstract of 541 articles (after duplicates were removed) were screened by two independent reviewers and excluded for analysis if they (1) were not a primary research article; (2) were a case report; (3) did not examine renal physiological response (hemodynamic regulation and/or electrolyte balance) to high altitude; (4) examined renal physiological response, but in an existing medical condition or pathophysiological condition (i.e., not the healthy kidney model); (5) examined renal physiological response, but under intermittent exposure to high altitude (i.e., not continuous); (6) examined the genetic change underlying the renal physiological response; (7) examined the effect of exercise training on renal physiological response; (8) examined the effect of treatment on renal physiological response; or (9) full article not available (e.g., supplement). Any discrepancy between the two independent reviewers was resolved by discussion. In total, 32 articles were retrieved for full article review, of which 14 were included in the final review. The article selection process is shown in Figure 1.

## Results

**Figure 2:**
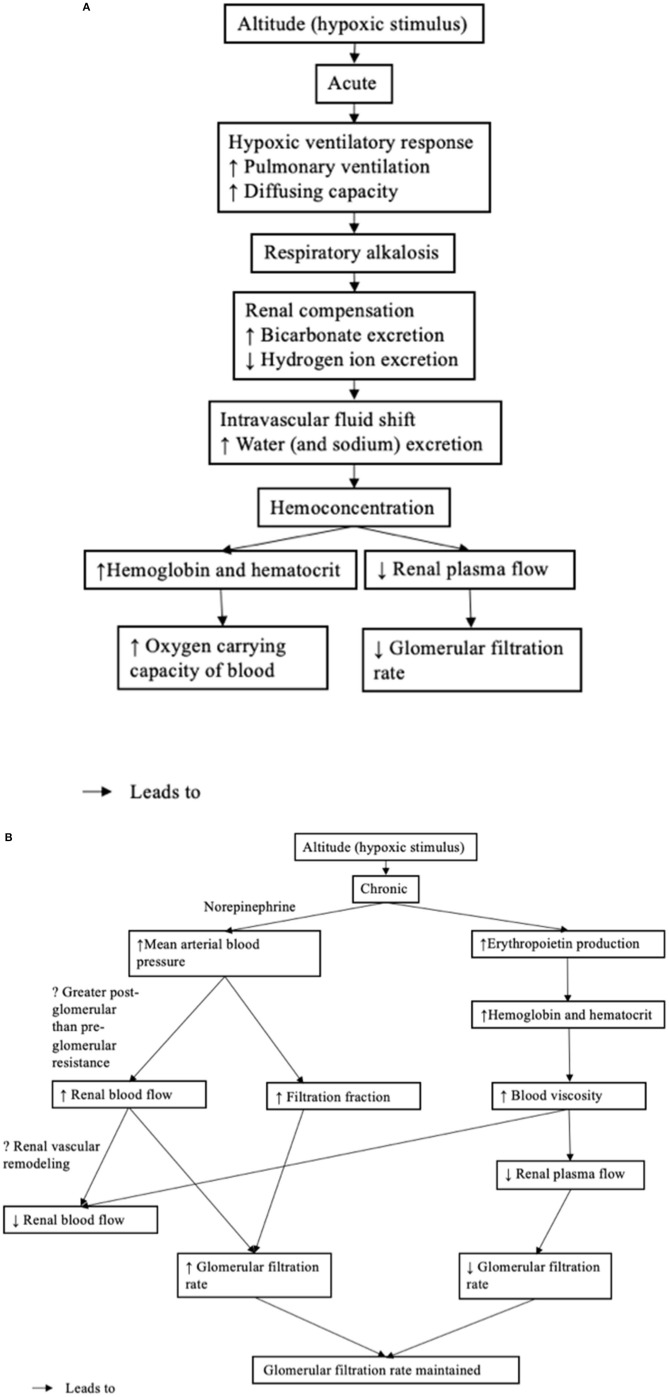
**(A)** Acute renal physiological response to high altitude. **(B)** Chronic renal physiological response to high altitude.

Fourteen articles were included in the review (shown in Table 1). Renal tissue adaptation to altitude-induced hypoxia was either investigated in a rat model (Thron et al., 1998; Al-Hashem et al., 2012 or human model Lewis et al., 1997; Bestle et al., 2002; Cumbo et al., 2002, 2015; Ge et al., 2002; Jefferson et al., 2002; Singh et al., 2003; Loeppky et al., 2005; Haditsch et al., 2007, 2015; Pichler et al., 2008; Zouboules et al., 2018). Duration of exposure to high altitude ranged from 8 h (Loeppky et al., 2005) to 90 d (Al-Hashem et al., 2012), with an altitude up to 5,800 m above sea level (Singh et al., 2003). For organization, the results are grouped by hemodynamic (and hematological) effects, blood pressure and regulating hormones, electrolyte balance, and renal tissue oxygen. An overview of the results is shown in Figure 2.

**Table 1:** Sample characteristics, primary outcomes, conclusions, and strengths and limitations of included articles.

**Study**	**Sample characteristics**	**Primary outcomes**	**Conclusion**
Chronic exposure of rats to native high altitude increases in blood pressure via activation of the renin-angiotensin- aldosterone system Al-Hashem et al. (2012)	*N* = 20 Adult Wistar male rats LA group, *n* = 10 HA group, *n* = 10 Duration of exposure to hypoxia, days: 90 Altitude, meters above sea level: 2,800–3,150	*HA group compared to LA group:* Hct: Increased, *p* = 0.002 MAP: Increased, *p* = 0.001 Plasma renin activity: Increased, *p* = 0.0001 Plasma aldosterone: Increased, *p* = 0.0001 Plasma vasopressin: Increased, *p* = 0.001 Plasma norepinephrine: Increased, *p* = 0.0001 Plasma creatinine: NS Serum Na: Increased, *p* = 0.008 Urinary Na: Decreased, *p* = 0.005 Serum K: NS Urinary K: NS FENa: Decreased, *p* = 0.006 FEK: NS	Long-term exposure to high altitude resulted in elevated blood pressure, induced by the renin- angiotensin- aldosterone system activation.
Prolonged hypobaric hypoxaemia attenuates vasopressin secretion and renal response to osmostimulation in men Bestle et al. (2002)	*N* = 8 Persons Male, percentage: 100 Age, years (mean, range): 26, 22–32 Duration of exposure to hypoxia, days: 8 Altitude, meters above sea level: 4,559 Investigation: Hypertonic saline infusion (5%, 3.6 mL/kg) given on Day 6	*Days at HA compared to baseline (sea level) measure:* Oxygen saturation, Days 1–3 and 6–8: Decreased, *p* < 0.05 Body weight, Day 8: Decreased, *p* < 0.05 MAP, Days 1–3 and 6–8: Increased, *p* < 0.05 Plasma osmolality, Days 3 and 6–8: Increased, *p* < 0.05 Plasma vasopressin, Days 1–3 and 6–8: Decreased, *p* < 0.05 Plasma renin, Days 1–3 and 6–8: Decreased, *p* < 0.05 Plasma ANP: NS Plasma aldosterone, Day 3, 7, and 8: Decreased, *p* < 0.05 Hemoglobin, Days 1–3, 6, and 8: Increased, *p* < 0.05	Hypobaric hypoxemia resulted in hypovolemia and hyperosmolality due to suppression of vasopressin.
		Plasma Na, Days 1–3: Decreased, *p* < 0.05 Plasma K, Day 1: Decreased, *p* < 0.05 Plasma K, Day 8: Increased, *p* < 0.05 Plasma creatinine, Days 2, 3, and 6–8: Increased, *p* < 0.05 Plasma albumin: NS Na excretion rate, Day 6–8: Increased, *p* < 0.05 K excretion rate, Days 2 and 3: Decreased, *p* < 0.05 Creatinine clearance, Day 7: Decreased, *p* < 0.05 Urine osmolality, Days 1–3, and 6: Decreased, *p* < 0.05	
Acute mountain sickness, dehydration, and bicarbonate clearance: preliminary field data from the Nepal Himalaya Cumbo et al. (2002)	*N* = 115 Persons Male, percentage: 82.6 Age, years: not reported Control group, *n* = 30 persons Altitude group, *n* = 85 persons Altitude, meters above sea level: 1,400 Days to ascent, percentage: 1 (49.4) 2 (43.5) 3 (7.06) Altitude, meters above sea level: 2,050–4,350	*Altitude group, compared to control group:* Oxygen saturation: Decreased, *p* < 0.001 Rate of ketonuria: Increased, *p* = 0.014 Urine specific gravity: Increased, *p* = 0.037 *LLS, correlation:* Oxygen saturation: *r* = −0.25, *p* = 0.020 Urine specific gravity: *r* = 0.22, *p* = 0.043 Urine pH: *r* = −0.22, *p* = 0.04 *Urine pH, correlation:* Urine specific gravity: *r* = −0.55, *p* < 0.001 Oxygen saturation: NS	Increased LLS (indicating greater risk of high altitude illness) associated with increased urine specific gravity (dehydration), low oxygen saturation (hypoxaemia), and acidic urine.
Higher venous bicarbonate concentration associated with hypoxemia, not acute mountain sickness, after ascent to moderate altitude Cumbo et al. (2015)	*N* = 52 Persons Male, percentage: 86.5 Age, years (mean ± SD): 27.5 ± 8.3 Days to ascent, percentage: 1 (71.7) 2 (15.2) 3 (10.9) 4 (0.02) Altitude, meters above sea level: 2,000 −4,250	*Oxygen saturation, correlation:* Serum venous bicarbonate concentration: Inversely correlated, *p* < 0.001 Base excess: Inversely correlated, *p* < 0.001 *Venous bicarbonate anion concentration:* Base excess: *r* = 0.97, *p* < 0.001 *AMS score, correlation:* Venous bicarbonate anion concentration: NS Base excess: NS *Rate of ascent, correlation:* Venous bicarbonate anion concentration: NS Base excess: NS *LLS, correlation:* Venous bicarbonate anion concentration: NS Base excess: NS	Markers of serum bicarbonate anion retention is associated with decreasing oxygen saturation; however, is not associated with LLS or presence of AMS.
Determinants of erythropoietin release in response to short-term hypobaric hypoxia Ge et al. (2002)	*N* = 48 Persons Male, percentage: 66.7 Age, years (mean ± SD): 21 ± 2.5 Duration of exposure to hypoxia, hours at each altitude: 6 and 24 Altitude (simulated), meters above sea level: 1,780, 2,085, 2,454, and 2,800	*EPO concentration compared to baseline:* 6 h at 1,780, 2,085, 2,454, and 2,800 m: Increased, *p* < 0.05 *EPO concentration compared to lower altitudes:* 24 h at 2,454 and 2,800 m: Increased, *p* < 0.05 *Urine PO_2_ compared to baseline:* 6 h at 1,780, 2,085, 2,454, and 2,800 m: Decreased, *p* < 0.05 24 h at 2,454, and 2,800 m: Decreased, *p* < 0.05 *SaO_2_ compared to baseline:* 6 h at 1,780, 2,085, 2454, and 2,800 m: Decreased, *p* < 0.05 24 h at 2,454, and 2,800 m: Decreased, *p* < 0.05 *RBF compared to baseline:* 6 h at 1,780, 2,085, 2,454, and 2,800 m: NS	The increase in EPO is dose-dependent, with the threshold for sustained EPO release ≥2,100–2,500 m in most individuals. At the lowest altitude, short-term acclimatization restored renal tissue oxygenation and restrained the increase in EPO.
Renal adrenomedullin and high altitude diuresis Haditsch et al. (2007)	*N* = 33 Persons Trekking team: *n* = 17 persons Male, percentage: 53 Age, years (range): 19–65 Climbing team (flew to altitude): *n* = 16 persons Male, percentage: 75 Age, years (range): 23–63 Altitude, meters above sea level: 150 (LA), 3,440 (MA), 5,050 (HA) Duration of exposure, weeks: 4	*Variables compared to LA (combined trekking and climbing team):* Fluid loss, MA: Increased, *p* < 0.05 Fluid loss, HA: Increased, *p*< *p* < 0.005 Sodium loss, MA: NS Sodium loss, HA: Increased, *p* < 0.005 Urinary osmolality, MA and HA: Decreased, *p* < 0.005 Urinary creatinine excretion, MA and HA: Decreased, *p* < 0.005 Total plasma protein, MA: Increased, *p* < 0.05 Total plasma protein, HA: Increased, *p* < 0.005 Plasma Na concentration, MA and HA: NS Plasma osmolality, MA and HA: Decreased, *p* < 0.05 Urinary AM excretion, HA: Increased, *p*< *p* < 0.005 Plasma AM concentration, MA and HA: Increased, *p* < 0.005 *Urinary AM excretion, correlation:* Nocturnal diuresis: *r* = 0.72, *p* < 0.005 Natriuresis: *r* = 0.57, *p* < 0.005 Urinary osmolality: *r* = 0.88, *p* < 0.005	Increased renal AM production as a potential role in the diuretic and naturietic response to high altitude.
Volume regulation and renal function at high altitude across gender Haditsch et al. (2015)	*N* = 28 Persons Male, percentage: 71 Age, years (range): 19–65 Altitude and duration of exposure, meters above sea level: 3,440 (HA-1, Day 3) 5,050 (HA-2, Day 14)	*Selected urinary parameters:* HA-1 vs. SL: 24 h amount (male and female): Increased, *p* < 0.05 Creatinine (male): Decreased, *p* < 0.05 Osmolality (male and female): Decreased, *p* < 0.05 FENa: NS HA-2 vs. SL: 9 h amount (male): Increased, *p* < 0.05 24 h amount (male and female): Increased, *p* < 0.05	Irrespective of sex, mean 24-h urine production increased, with no change observed in plasma concentration, urinary concentration, or fractional excretion of Na. The above corresponded to decreased urinary osmolality. In addition, there was no change in aldosterone, and ANP increased only in male sex.
		Sodium (male): Increased, *p* < 0.05 Osmolality (male): Decreased, *p* < 0.05 FENa: NS HA-2 vs. HA-1: 9 h amount (male): Increased, *p* < 0.05 Sodium (male): Increased, *p* < 0.05 FENa: NS *Selected plasma parameters:* HA-1 vs. SL: ADH (male): Decreased, *p* < 0.05 Renin activity (male): Decreased, *p* < 0.05 ANP (male): Increased, *p* < 0.05 HA-2 vs. SL: Total protein (female): Increased, *p* < 0.05 Albumin (female): Increased, *p* < 0.05 Creatinine (male and female): Increased, *p* < 0.05 ADH (male and female): Decreased, *p* < 0.05 Renin activity (male and female): Decreased, *p* < 0.05 ANP (male): Increased, *p* < 0.05 *Selected GFR estimates:* HA-1 vs. SL: Creatinine/9 h (male and female): Decreased, *p* < 0.05 CKD-EPI (male): Decreased, *p* < 0.05 HA-2 vs. SL: Creatinine/9 h (male and female): Decreased, *p* < 0.05 MDRD (male and female): Decreased, *p* < 0.05 Mayo (male and female): Decreased, *p* < 0.05	
Hyperuricemia, hypertension, and proteinuria associated with high-altitude polycythemia Jefferson et al. (2002)	*N* = 80 Persons Male, percentage: 100 Control group (SL), *n* = 28 Control group at (SA) altitude, meters above sea level: 4,300 Control group, duration of exposure: 48 h Group 1 (HA, Hct < 65%), *n* = 25 Group 2 (EE, Hct > 65%), *n* = 27 Altitude, meters above sea level: 4,300 Duration of exposure: Living at HA	*HA compared to SL:* Mean blood pressure: NS 24 h urine sodium: NS Fractional lithium excretion: *p* < 0.0001 Fractional sodium excretion: Increased, *p* = 0.02 Renin mass: NS Creatinine clearance: NS 24hr urine protein: NS *EE compared to SL:* Mean blood pressure: Increased, *p* < 0.0001 24 h urine sodium: Increased, *p* = 0.02 Fractional lithium excretion: Decreased, *p* < 0.0001 Fractional sodium excretion: Increased, *p* < 0.0001	Response to high altitude indicates increased proximal reabsorption of filtrate. Renal function was normal; however, proteinuria was greatest in EE group.
		Renin mass: Decreased, *p* = 0.0008 Creatinine clearance: NS 24 h urine protein: Increased, *p* = 0.003 *EE compared to HA:* Mean blood pressure: Increased, *p* = 0.005 24 h urine sodium: NS Fractional lithium excretion: Decreased, NS Fractional sodium excretion: Increased, *p* = 0.02 Renin mass: Decreased, *p* = 0.02 Creatinine clearance: NS 24 h urine protein: Increased, *p* = 0.01 *Hct correlation:* CMS score: *r* = 0.67, *p* < 0.001 SaO_2_: *r* = −0.88, *p* < 0.001 Urine total protein: *r* = 0.25, *p* = 0.005	
Capillary filtration coefficient and urinary albumin leak at altitude Lewis et al. (1997)	*N* = 8 Persons Male, percentage: 75 Age, years (range): 25–54 Altitude and duration of exposure, meters above sea level: 1,500 (5.75 h) 4,559 (23.5 h)	*CFC compared to baseline:* 5.75 h: NS 23.5 h: Increased, *p* < 0.02 *Urinary albumin concentration, mg per mmol of creatinine:* 5.75 h: NS 23.5 h: Increased, *p* < 0.05 *MAP compared to baseline:* 5.75 h: NS 23.5 h: Increased, *p* < 0.02	No significant association between CFC (absolute and relative change), AMS questionnaire score, urinary albumin concentration, or change in MAP.
Early fluid retention and severe acute mountain sickness Loeppky et al. (2005)	*N* = 51 Persons AMS group (*post-hoc*), *n* = 16 Non-AMS group (*post- hoc*), *n* = 16 Altitude (simulated), meters above sea level = 4,800 Duration of exposure to altitude, hours (range): 8–12	*AMS and non-AMS combined at 12 h compared to baseline:* Fluid intake: Decreased, *p* < 0.001 Urine volume: Decreased, *p* < 0.001 Net fluid balance: NS GFR: NS Plasma K: NS Plasma Na: Decreased, *p* = 0.041 Urine Na/K: NS K excretion: Decreased, *p* = 0.025 Na excretion: Decreased, *p* < 0.001 PV: NS ANP: NS Plasma renin activity: NS Aldosterone: Decreased, *p* < 0.001 AMS: Increased, *p* < 0.001 Free water clearance; Decreased, *p* = 0.001 ADH: Increased, *p* = 0.022	Excretion of Na and K decreased at altitude, but with no significant difference (overall) between AMS and non-AMS group. Exposure to altitude had no appreciable effect on plasma renin activity.
Glomerular filtration rate estimates decrease during high altitude expedition but increase with Lake Louise acute mountain sickness score Pichler et al. (2008)	*N* = 27 Persons: Group 1 (faster ascent/shorter acclimatization period), *n* = 14 Male, percentage: 85.7 Age, years (mean ± SD): 44.4 ± 13.4 Duration of exposure, days: 9 Group 2 (slower ascent/longer acclimatization period), *n* = 13 Male, percentage: 76.9 Age, years (mean ± SD): 44.6 ± 7.9 Duration of exposure, days: 13 Altitude, meters: 450 (Pre-expedition) 4,497 (Base Camp) 5,533 (Camp 1) 6,265 (Camp 2) 6,865 (Camp 3)	*Group 2, compared to Group 1:* eGFRCYS: NS eGFRMAYO: NS eGFRMDRD: NS *eGFR changes during high altitude expedition:* eGFRCYS: Decreased, *p* = 0.02 eGFRMAYO: NS eGFR_MDRD_: Not reported *AMS, correlation:* eGFRCYS: *r* = 0.28, *p* = 0.01 eGFRMAYO: NS eGFR_MDRD_: Not reported *Hct, correlation:* eGFRCYS: *r* = −0.28, *p* = 0.01 eGFRMAYO: NS eGFR_MDRD_: Not reported	Renal function declined with increasing altitude, as demonstrated by decreased eGFR (cys), but increased with AMS score.
		*Hormonal changes during high altitude expedition:* Aldosterone: Increased, *p* = 0.017 Renin concentration: NS Aldosterone-renin ratio: NS BNP: NS *eGFRCYS, correlation:* Aldosterone: NS Renin concentration: NS Aldosterone-renin ratio: NS BNP: NS	
Blood gases, hematology, and renal blood flow during prolonged mountain sojourns at 3,500 and 5,800 m Singh et al. (2003)	*N* = 15 persons Male, percentage: 100 Age, years (mean ± SD): 22 ± 2.3 Altitude and duration of exposure, meters above sea level: 260 (sea level) 3,500 (60 d stay) 5,800 (70 d stay)	*3,500 m at 60 d compared to sea level:* pH: Increased, *p* < 0.001 PaO_2_: Decreased, *p* < 0.001 PaCO_2_: Decreased, *p* < 0.001 Hematocrit, Increased, *p* < 0.001 Hemoglobin: Increased, *p*< *p* < 0.001 Blood viscosity: Increased, p < 0.001 Effective RBF: Decreased, *p* < 0.001 Effective RPF: Decreased, p < 0.001 Plasma Na: NS Plasma K: Increased, *p* < 0.05 *5,800 m at 70 d compared to sea level:*	At moderate altitude, blood pH increased and remained increased at high altitude. PaO_2_ was decreased by 39%, while blood viscosity was increased by 38% at high altitude. Effective renal plasma flow was significantly reduced.
		pH: Increased, *p* < 0.001 PaO_2_: Decreased, *p* < 0.001 PaCO_2_: Decreased, *p* < 0.001 Hematocrit, Increased, *p* < 0.001 Hemoglobin: Increased, *p* < 0.001 Blood viscosity: Increased, *p* < 0.001 Effective RBF: Decreased, *p* < 0.001 Effective RPF: Decreased, *p* < 0.001 Plasma Na: Increased, *p* < 0.001 Plasma K: Increased, *p* < 0.05	
Renovascular adaptive changes in chronic hypoxic polycythemia Thron et al. (1998)	*N* = 65 Male Sprague- Dawley rats Hilltop (develop excessive polycythaemia), *n* = 32 Madison (develop moderate polycythaemia), *n* = 33 Duration of exposure: 0, 1, 3, 14, and 30 d Altitude (simulated), atm: 0.5	*Change as the function of hematocrit:* RBF: Slope = 91.2 ± 8.8 μl/min/100 g body wt/% Hct, *p* < 0.001 FF at a hematocrit of 70%: Slope = 0.0036–0.0010, *p* < 0.002 RPF: Slope = −17.2 ± 4.3 μl/min/100 g body wt/% Hct, *p* < 0.001 GFR: Slope = −3.3 ± 2.2 μl/min/100 g body wt/% Hct, *p* = 0.17 *Hilltop compared to Madison:* RBF: NS FF: NS RPF: NS GFR: NS *Renal oxygen delivery under hypoxic condition:* Hilltop, slope: 1.17 ± 0.08 ml/min/100 g body wt/% Hct, *p* < 0.001 Madison, slope: 1.59 ± 0.20 ml/min/100 g body wt/% Hct, *p* < 0.001 Hilltop vs. Madison: *p* = 0.04 *Renal oxygen consumption (normoxic and hypoxic combined):* Hilltop, slope: 0.077 ± 0.028 mol/min/100 g wt/% Hct, *p* < 0.02 Madison, slope: 0.071 ± 0.038 mol/min/100 g wt/% Hct, *p* < 0.07 Hilltop vs. Madison: *p* < 0.01	Haematocrit exceeded the sea level control value by 50% at peak haematocrit. FF increased with hematocrit, RPF decreased, and GFR remained constant.
Renal reactivity: acid-base compensation during incremental ascent to high altitude Zouboules et al. (2018)	*N* = 20 persons Male, percentage: 50 Age, years (mean ± SD): 27.5 ± 9.5 Altitude and duration of exposure, meters above sea level: 1,045 or 1,400 (base line) 3,440 (Day 3) 3,820 (Day 5) 4,240 (Day 7) 5,160 (Day 10)	PaO_2_: Decreased, *p* < 0.001 PaCO_2_: Decreased, *p* < 0.001 Bicarbonate: Decreased, *p* < 0.001 Hematocrit: Increased, *p* < 0.001 Creatinine: NS Osmolality: Decreased, *p* < 0.05	Hypoxia and hypocapnia were seen with ascent to altitude. The decrease in bicarbonate, in part, confirmed the relative compensatory metabolic acidosis. Renal compensation plateaued after day 5, despite further increase in altitude.

*ADH, Antidiuretic hormone; AM, Adrenomedullin; AMS, Acute mountain sickness; ANP, Atrial natriuretic peptide; BNP, Brain natriuretic peptide; CFC, Capillary filtration capacity; CH_2_O, Free water clearance; CKD-EPI, Chronic Kidney Disease Epidemiology Collaboration; DBP, Diastolic blood pressure; EE, Excessive erythrocytosis; EPO, Erythropoietin; FEK, Fractional excretion of potassium; FENa, Fractional excretion of sodium; GFR, Glomerular filtration fraction; HA, High altitude; Hb, Hemoglobin; Hct, Hematocrit; K, Potassium; LA, Low altitude; LL, Lake Louise; LLS, Lake Louise score; MA, Medium altitude; MAP, Mean arterial pressure; MDRD, Modification of Diet in Renal Disease; Na, Sodium; NS, Not significant; PaCO_2_, Partial pressure of arterial carbon dioxide; PaO_2_, Partial pressure of arterial oxygen; PV, Plasma volume; RBF, Renal blood flow; RPF, Renal plasma flow; SaO_2_, Arterial oxygen saturation; SD, Standard deviation; SBP, Systolic blood pressure*.

### Hemodynamic (and Hematological) Effects

Six studies examined erythropoietin, hematocrit, hemoglobin, and/or blood viscosity (Thron et al., 1998; Bestle et al., 2002; Ge et al., 2002; Singh et al., 2003; Al-Hashem et al., 2012; Zouboules et al., 2018). At an altitude above 2,100 and 2,500 m, erythropoietin production was increased and sustained at 24 h (Ge et al., 2002). At a lower altitude, the change in erythropoietin was transient (i.e., not sustained after 24-h exposure). However, substantial inter-individual variability was noted. Similar to erythropoietin, hematocrit was increased at chronic exposure (up to 90 d) to high altitude, which ranged from 2,800 to 5,800 m (Thron et al., 1998; Singh et al., 2003; Al-Hashem et al., 2012; Zouboules et al., 2018). The increase in hematocrit was accompanied by an increase in hemoglobin and blood viscosity (Bestle et al., 2002; Singh et al., 2003).

Five studies examined the hemodynamic change to high altitude, including glomerular filtration rate (Thron et al., 1998; Bestle et al., 2002; Jefferson et al., 2002; Loeppky et al., 2005; Pichler et al., 2008; Haditsch et al., 2015), renal blood flow (Thron et al., 1998; Singh et al., 2003), and renal plasma flow (Thron et al., 1998; Singh et al., 2003). Two studies found no significant change in glomerular filtration rate at 8–12 h (at 4,800 m) and at 48 h (at 4,300 m) (Jefferson et al., 2002; Loeppky et al., 2005); however, there was a tendency to decrease noted in one study. In another study, glomerular filtration rate decreased with ascent to high altitude; however, at a higher altitude (from 4,497 to 6,865 m) decreased glomerular filtration rate was demonstrated only by cystatin C and not by creatinine clearance (Pichler et al., 2008). In another study, glomerular filtration rate (indicated by creatinine clearance) decreased and reached significance at day 3; however, it was normalized at day 7 (Bestle et al., 2002). In the last study, glomerular filtration rate (indicated by absolute renal creatinine), decreased at exposure to 3,440 m (day 3); however, it remained stable at exposure to 5,050 m (day 14) (Haditsch et al., 2015).

Renal blood flow increased as hematocrit increased in one study; however, the increase in renal blood flow was not enough to maintain renal plasma flow and thus, renal plasma flow decreased (at 30 days exposure to a simulated altitude of 0.5 atm, equivalent to an altitude >4,500 m) (Thron et al., 1998). In another study, at relatively longer exposure to high altitude, resulted in decreases in both renal blood flow and renal plasma flow (70 d exposure to 5,800 m) (Singh et al., 2003). Despite the decreased renal plasma flow, glomerular filtration rate was maintained in one study by the increased filtration fraction (and increased renal blood flow) (Thron et al., 1998) and not studied in the second study (Singh et al., 2003). In both, the blood viscosity increased. Thron et al. (1998) concluded that the increase in renal blood flow also corresponded to a decrease in the renal vascular resistance and hindrance (resistance/viscosity).

To summarize, sustained erythropoietin production was observed only at an altitude >2,100 m and driven largely by the magnitude of the hypoxic stimulus. At a lower altitude, it is unclear whether the duration alone is enough to drive a sustained increase in erythropoietin production. Similarly, hematocrit, hemoglobin, and blood viscosity increased at exposure to high altitude. Again, the impact of duration of exposure on the hematocrit, hemoglobin, and blood viscosity is unclear. At exposure to high altitude, an initial decrease in glomerular filtration rate that became significant after 48 h was observed. It is unclear whether the increase in erythropoietin production, hematocrit, hemoglobin, and blood viscosity caused the initial decrease in glomerular filtration rate. At 1 week, glomerular filtration rate returned to normal, which could be explained by an increase in filtration fraction. Alternatively, renal blood flow increased as renal vascular resistance decreased, which could suggest another mechanism (e.g., renal vascular remodeling).

### Blood Pressure and Regulating Hormones

Blood pressure (systolic, diastolic, and/or mean arterial pressure) and regulating hormones were examined in six studies (Thron et al., 1998; Bestle et al., 2002; Jefferson et al., 2002; Loeppky et al., 2005; Al-Hashem et al., 2012; Haditsch et al., 2015). When exposed to acute (1–3 d at 4,559 m) and chronic (90 d at 2,800 to 3,150 m) high altitude, blood pressure increased in two studies (Bestle et al., 2002; Al-Hashem et al., 2012). However, in another study, blood pressure increased only in the group at high altitude with excessive erythrocytosis (defined by hematocrit >65%) (Jefferson et al., 2002). Increased blood pressure was not observed in the group with normal hematocrit (equal to or <65%) (Jefferson et al., 2002). In the last study, blood pressure remained unchanged during exposure to chronic (30 d) high altitude and no difference was observed between rat strains with different polycythemic responses to high altitude (Thron et al., 1998).

Increased blood pressure was associated with increased renin plasma activity at 90 d exposure to high altitude (2,800–3,150 m) (Al-Hashem et al., 2012). However, plasma renin activity was unchanged in one study at 8–12 h (Loeppky et al., 2005), decreased in two other studies at 1–3 d and at 4 w (Bestle et al., 2002; Haditsch et al., 2015), and decreased only in the group with excessive erythrocytosis in another study at 48 h (Jefferson et al., 2002). The level of aldosterone was increased in one study at 90 d (Al-Hashem et al., 2012); however, it was decreased in two studies at 8–12 h and at 4 w (Loeppky et al., 2005; Haditsch et al., 2015) and decreased at day 3, but unchanged at days 1 and 2 in a third study (Bestle et al., 2002). Vasopressin remained either unchanged at 1–3 d (Bestle et al., 2002), decreased at 4 w (Haditsch et al., 2015), or increased at 8–12 h and at 90 d (Loeppky et al., 2005; Al-Hashem et al., 2012). Norepinephrine was increased in one study at 90 d (Al-Hashem et al., 2012); however, in a second study the increase became significant only at day 6 (Bestle et al., 2002).

To summarize, the relationship between high altitude and blood pressure is difficult to discern; however, blood pressure does appear to increase upon exposure to both acute and chronic high altitude. The increase in blood pressure might be dependent on whether or not erythropoietin production is sustained (i.e., presence of erythrocytosis), which could be a function of the extent (or magnitude) of altitude exposure. The relationship between blood pressure and regulating hormones is unclear; however, renin, aldosterone, vasopressin, and norepinephrine may play a role in the increase in mean arterial pressure at chronic exposure (90 d) to high altitude. At acute exposure to high altitude, the hormonal changes appeared to be more transient (i.e., oscillating between being unchanged, decreased, and increased).

### Electrolyte Balance

Eight studies examined electrolyte balance (Thron et al., 1998; Bestle et al., 2002; Jefferson et al., 2002; Singh et al., 2003; Loeppky et al., 2005; Haditsch et al., 2007, 2015; Al-Hashem et al., 2012). Four studies examined sodium excretion rate (Thron et al., 1998; Bestle et al., 2002; Loeppky et al., 2005; Haditsch et al., 2007), three studies examined fractional excretion of sodium (Jefferson et al., 2002; Al-Hashem et al., 2012; Haditsch et al., 2015), and one study examined only plasma (or serum) sodium (Singh et al., 2003). At exposure to high altitude, sodium excretion rate remained unchanged in two studies (at 0.5 atm over 30 d, which is equivalent to an altitude >4,500 m and at 4,559 m for 1–3 d) (Thron et al., 1998; Bestle et al., 2002), decreased in one study (at 4,800 m for 8–12 h) (Loeppky et al., 2005), and increased in one study (at 5,050 m at day 14) (Haditsch et al., 2007). Fractional excretion of sodium either remained unchanged at 3,400 m (at day 3) and 5,050 m (at day 14) or decreased at 2,800–3,150 m (at 90 d) and 4,300 m (at 48 h) (Jefferson et al., 2002; Al-Hashem et al., 2012).

To summarize, the sodium excretion rate and fractional excretion of sodium at exposure to high altitude is inconsistent. No clear relationship between sodium excretion rate, fractional excretion of sodium, and duration and extent of exposure to high altitude was evident. This might suggest that the renal excretory response to a decrease in the partial pressure of atmospheric oxygen is in a constant state of change, dynamically adjusting to maintain plasma sodium and electrolyte homeostasis at high altitudes.

### Renal Tissue Oxygen

Three studies examined renal tissue oxygen, a function of both renal oxygen delivery and consumption (Thron et al., 1998; Ge et al., 2002; Singh et al., 2003). In one study, arterial partial pressure of oxygen (PaO_2_), an indicator of renal oxygen delivery, decreased at high altitude (at 5,800 m at 70 d) (Singh et al., 2003). Another study examined urine PO_2_, an estimate of renal tissue oxygenation (Ge et al., 2002) and found that at 6 h, urine PO_2_ decreased at all altitudes; however, returned to baseline by 24 h at the lowest altitude (1,780–2,085 m), but not at the highest altitude (2,454–2,800 m) (Ge et al., 2002). Despite individual variability, the mean urine PO_2_ was significantly correlated with the mean blood erythropoietin level. The best estimate of renal tissue oxygen was provided by Thron et al. (1998) who calculated renal oxygen delivery and consumption from renal blood flow and arterial oxygen content or renal arteriovenous oxygen content difference, respectively. Both renal oxygen delivery and consumption increased. Furthermore, renal oxygen delivery increased as a function of hematocrit. Only a minor change was noted in sodium balance (filtered sodium load, fractional sodium reabsorption, and sodium excretion) and the authors could not explain the increase in renal oxygen delivery. Unfortunately, the data were not provided.

To summarize, renal oxygenation might decrease initially, but at a lower altitude is able to return to baseline. At a higher altitude, arterial PaO_2_ was decreased, but hematocrit and hemoglobin are both increased facilitating tissue oxygenation (consistent with erythropoietin production being sustained at the higher altitude). Instead, this could reflect a decrease in oxygen delivery as a result of decreased blood flow caused by increased blood viscosity. Arterial PaO_2_ alone is a poor measure of oxygen delivery, because it does not account for the change in renal blood flow and renal plasma flow. When renal blood flow was used to calculate renal oxygen delivery, renal oxygen delivery increased as a function of hematocrit. Unfortunately, no study examined renal oxygenation in the context of the tubular transport of sodium or the metabolic efficiency of the tubular transport of sodium, which could give great insight into the impact of high altitude on the efficiency of renal oxygen consumption.

### Acute Mountain Sickness

Five studies examined characteristics associated with acute mountain sickness (AMS; defined by the Lake Louise criteria) (Lewis et al., 1997; Cumbo et al., 2002, 2015; Loeppky et al., 2005; Pichler et al., 2008), including measures of acid/base balance (Cumbo et al., 2002, 2015; Loeppky et al., 2005), fluid balance and free water clearance (Cumbo et al., 2002; Loeppky et al., 2005), electrolyte plasma concentration and excretion (Loeppky et al., 2005), regulating hormone concentration (e.g., aldosterone, atrial natriuretic peptide, and plasma renin activity) (Loeppky et al., 2005), hematological factors (Pichler et al., 2008), and indicators of renal injury (Lewis et al., 1997). Three studies were performed in an altitude chamber (Thron et al., 1998; Ge et al., 2002; Loeppky et al., 2005). Understanding the underlying pathophysiology of AMS was not an objective of the current review and is not discussed.

### Limitations

The body of research, as a whole, has a number of limitations that should be considered when interpreting the results. First, the lack of the well-controlled environment (e.g., temperature, pressure, diet, and exercise) is a limitation in the field trials and is difficult to account for in the results. Second, most of the field trials did not control and/or measure dietary salt and fluid intake, which can make it difficult to compare results. The use of an altitude chamber, on the other hand, permitted the environment to be controlled. However, the use of a normobaric hypoxic chamber can make comparison to either a hypobaric hypoxic chamber or field trial more difficult. Third, the variation in the measurement of renal function can also make it difficult to compare results (e.g., inulin clearance, creatinine clearance, and serum creatinine). Despite inulin clearance being an accurate measure of glomerular filtration rate, the administration of an exogenous marker may not be practicable on a mountain expedition at high altitude. Creatinine clearance is subject to exercise-induced release and is, again, difficult to account for in the results. Lastly, most of the studies that used a human model did not report baseline characteristics including race, body weight, smoking status, or whether participants used a carbonic anhydrase inhibitor to prevent acute mountain sickness.

## Discussion

The systematic review examined the effect of high altitude on renal physiological function (hemodynamic regulation and electrolyte balance). An overview of the renal physiological response is found in Figures 2A,B. It is important to note, that the renal physiological response is part of a larger response, coordinated with the respiratory and cardiovascular systems. The overall purpose of the integrated responses is to maintain tissue oxygenation and in the case of the kidney, to maintain acid-base balance, with challenges to renal tissue oxygenation.

In response to high altitude, there is a well-described hypoxic ventilatory response whereby tissue oxygenation is improved by increased pulmonary ventilation, leading to a respiratory alkalosis (Goldfarb-Rumyantzev and Alper, 2014). The renal system compensates for the alkalosis by excreting excess bicarbonate and conserving hydrogen ions. At an altitude <2,100 m, this initial response appears to be completed within 24 h; however, at a higher altitude, urine pH remained increased in one study suggesting that bicarbonate excretion is incomplete (Ge et al., 2006). It is clear that the extent (or magnitude) of altitude does have an effect on the diuresis, but what is unclear is whether the effect is the result of the respiratory alkalosis alone or whether hypoxia is involved in the mechanism underlying the diuresis.

Further to the bicarbonate diuresis, there is a shift in the intravascular fluid and an increase in the water (and sodium) excretion. This is referred to as the diuretic and natriuretic response and, again, appears to be triggered within hours of exposure to high altitude, followed by return to baseline with normal renal sodium and fluid excretion rate (Goldfarb-Rumyantzev and Alper, 2014). Further, the initial diuretic-response occurs in the absence of the natriuretic-response, although the mechanism is unknown (Goldfarb-Rumyantzev and Alper, 2014). Consistent with this, in one study, the 24-h urine production had increased (and was accompanied by weight loss); however, the plasma concentration, urinary concentration, and fractional excretion of sodium had not changed at exposure to 3,440 m (on Day 3 of the ascent) or 5,050 m (on Day 14 of the ascent) (Haditsch et al., 2015). Similarly, in a second study, the plasma osmolality increased (and was accompanied by weight loss) at exposure to 4,559 m (Bestle et al., 2002). Anti-diuretic hormone (vasopressin) was decreased in both studies, suggesting that hypoxia might attenuate vasopressin secretion, which could prove to be the underlying mechanism. Bestle et al. (2002) concluded that exposure to hypoxia can elevate the set point of plasma osmolality:plasma vasopressin relationship. Again, the degree of exposure to high altitude affects the diuresis, but what remains unclear is the effect of duration on the diuresis and whether at the higher altitude (>2,100 m) the increase in renal fluid excretion returns to baseline. No clear relationship could be discerned between sodium excretion rate and the extent and duration of exposure to high altitude. However, a relationship might emerge due to regulating hormones including renin, aldosterone, vasopressin, and norepinephrine.

The diuretic response to high altitude and resulting depletion in the circulating volume, led both Bestle et al. (2002) and Haditsch et al. (2015) to postulate the presence of hemoconcentration. They suggest that hemoconcentration could underlie the initial increase in hemoglobin (and hematocrit) (Bestle et al., 2002). Bestle et al. (2002) report that hematocrit increased prior to the erythropoietic effect; however, it is unclear when the erythropoietic effect became significant. Hemoconcentration can serve to increase the oxygen-carrying capacity of blood and therefore may be adaptative. These data describe the role played by the kidney as an early blood/tissue oxygen sensor. The immediate response of the kidney is to increase the excretion of water, which concentrates the blood and increases hematocrit. Importantly this response is rapid in onset and it precedes erythropoiesis, which is dependent upon a slower genomic response.

In response to prolonged high altitude, erythropoietin production is increased (shown in Figure 2B). This is perhaps the most clear and consistent finding in the review of the literature. Erythropoietin production can serve to increase both red blood cell mass and hemoglobin concentration to improve the oxygen carrying capacity of the blood (Faura et al., 1969). Interestingly, exposure to an altitude >2,100 m was potent enough to cause a sustained increase in erythropoietin production Here, it is clear that erythropoietin production is related to the magnitude of the exposure to high altitude. Consistent with the increased erythropoietin production, both hemoglobin and hematocrit were increased at exposure to higher altitudes (>2,100 m); however, these were not studied at the lower altitude (Thron et al., 1998; Singh et al., 2003; Al-Hashem et al., 2012). It must also be stressed that the magnitude of the hypoxic stimulus is only related to erythropoietin expression through its impact upon cortico-medullary oxygen tension in the kidney. Importantly, a decrease in cortico-medullary oxygen tension is detected by the peritubular fibroblast cells, whose intracellular machinery transduces the signal into an upregulation in erythropoietin expression and secretion (Donnelly, 2001; Haase, 2010). At a lower altitude, atmospheric oxygen is not as low, raising the question as to whether long duration exposure to low/moderate degrees of atmospheric hypoxia is enough to drive a decrease in cortico-medullary oxygen tension potent enough to induce a sustained increase in erythropoietin production (or increase in hemoglobin and hematocrit).

At acute exposure to high altitude, there is an initial decrease in glomerular filtration rate (shown in Figure 2A). Although, only speculative, the initial decrease in glomerular filtration rate may be due to hemoconcentration and the corresponding increase in blood viscosity and decrease in renal plasma flow. At chronic (or prolonged) exposure to high altitude, the glomerular filtration rate appears to return to normal (shown in Figure 2B). In one study, the renal blood flow and filtration fraction increased to maintain the glomerular filtration rate in the presence of reduced renal plasma flow (Thron et al., 1998). While Thron et al. (1998) suggest that the increase in renal blood flow corresponded to a decrease in renal vascular resistance as a result of the dilation of the afferent and efferent arterioles, there is evidence to suggest that increased filtration fraction is caused by the greater increase in the post-glomerular resistance compared with the pre-glomerular resistance as a result of renal sympathetic nerve activity (Denton et al., 2002). Elevated renal sympathetic nerve activity is supported by the increase in circulating norepinephrine observed in one study, which became significant at day 6 and may indicate a chronic (rather than acute) mechanism to maintain glomerular filtration rate (Bestle et al., 2002). The decrease in renal vascular resistance observed by Thron et al. (1998) might better reflect vascular remodeling driven by chronically increased renal blood flow. Although only speculative, vascular enlargement might explain the decreased renal blood flow, despite elevated blood viscosity observed in the other study at a longer duration of exposure to high altitude (Singh et al., 2003).

It is clear that the magnitude and duration of exposure to high altitude affects glomerular filtration rate, renal blood flow, renal plasma flow, and filtration fraction; however, the relationship and mechanism underlying the relationship is still unclear, especially in the initial response when the myogenic and tubuloglomerular feedback mechanism might play a larger role. In any case, it does appear that glomerular filtration rate is maintained perhaps at the expense of renal blood flow. Further research into the mechanisms that are both intrinsic and extrinsic to the kidney is needed to better understand the renal physiological response to high altitude, including the mechanisms to prevent the vicious cycle between erythropoietin production, increased blood viscosity, decreased blood flow, and increased tissue hypoxia (Erslev et al., 1985).

The relationship between hematocrit and oxygen (O_2_) delivery is an inverted U-shaped relationship, where at low hematocrit the O_2_ delivery is decreased because of the limited oxygen-carrying capacity of blood and at high altitude where oxygen delivery is decreased because of decreased blood flow as a result of increased blood viscosity. This relationship was observed in one study whereby a highly significant curvilinear (inverted U-shaped relationship) was found between urine partial pressure of oxygen (PO_2_) and blood erythropoietin, suggesting that renal tissue PO_2_ may drive the erythropoietin production (Ge et al., 2002). This relationship was seen at acute exposure (24 h) to high altitude, but not at chronic exposure (30 d) to high altitude (Thron et al., 1998). The above might be explained by the fact that renal blood flow did not decrease as the hematocrit increased (despite increased blood viscosity) at chronic exposure to high altitude, possibly because of vascular remodeling or enlargement (Thron et al., 1998). Interestingly, increased blood pressure, which could offset the effect of increased blood viscosity on blood flow, was not increased in the study (Thron et al., 1998).

The regulation of blood pressure is a complex process. At exposure to high altitude, blood pressure was either unchanged or increased; however, this appeared to be dependent on the hematocrit. Although, only speculative, altered blood pressure might be dependent on whether or not erythropoietin production is sustained (i.e., presence of erythrocytosis), which may be a function of the extent of altitude exposure. However, no difference in systemic blood pressure was observed at chronic exposure to high altitude between rat strains with different polycythemic responses (Hilltop, which develops excessive polycythemia and Madison, which develops moderate polycythemia) (Thron et al., 1998), suggesting that increased hematocrit may not be driving the increase in blood pressure (at least at chronic exposure). Rather, a different mechanism may underlie the increase in blood pressure both at acute (1–3 d) and chronic (70 d) exposure to high altitude. At acute exposure to high altitude, the increase in blood pressure might also be mediated, in part, by an increase in the sympathetic drive. Interestingly, at acute exposure to altitude, blood pressure increased in one study (at 1–3 d), prior to when elevated blood norepinephrine became significant (at 6 d) (Bestle et al., 2002). However, in a prior study, systolic blood pressure increased with epinephrine on day 1, followed by an increase in diastolic and mean blood pressure as norepinephrine increased (Kanstrup et al., 1998). At chronic exposure to high altitude, renin, aldosterone, vasopressin, and norepinephrine might play a larger role. However, further research is needed to discern the role of renin, aldosterone, vasopressin and norepinephrine in relation to blood pressure control at high altitude.

Concerning electrolyte balance and renal oxygen tension, the data are limited and the evidence that is available is difficult to discern. Nevertheless, renal oxygenation may decrease initially, but return to baseline at lower altitudes. This came from evidence in one study, that showed that at 6 h, urine partial pressure of oxygen (PO_2_) decreased at all altitudes; however, it returned to baseline by 24 h at the lowest altitude (1,780–2,085 m), but not at the highest altitude (2,454–2,800 m) (Ge et al., 2002). Urine PO_2_ is an estimate of renal tissue oxygenation (or kidney tissue PO_2_), but more specifically it is an estimate of medullary PO_2_ and is indicative of medullary hypoxia. The medulla is particularly vulnerable to hypoxia because of low medullary blood flow. The mechanism underlying the improvement in urine PO_2_ at 24 h at the lower altitude is not known; however, it may relate to the initial hypoxic ventilatory response.

At exposure to chronic high altitude, Thron et al. (1998) observed an increase in both renal oxygen delivery and consumption. The study noted only a minor change in solute (sodium) exchange and could not explain the increased renal oxygen consumption. However, there is an increasing amount of evidence in the disease model to suggest that the change in renal oxygen consumption in the absence of an overt change in sodium excretion could reflect a decrease in the metabolic efficiency of sodium transport in the kidney (Hansell et al., 2013). As such, further research is needed to extend the experimental findings and examine in more detail the effect of exposure to high altitude on intrarenal oxygen consumption, the efficiency of sodium transport, and basal metabolism in the kidney using a direct method to assess renal tissue oxygen.

## Limitations

The review has several limitations to note: (1) Articles written before 1997 were excluded. The review focused on more recent evidence that looked at the complex system interaction and not at the individual components. (2) The review did not examine AMS and only considered the combined group (AMS and non-AMS) when possible. The pathophysiology underlying the development of AMS is multi-factorial and not well understood; it was not an objective of the review. (3) The review included articles that used the high altitude model to examine renal adaptation to hypoxia, thereby excluding other models and should be understood within the larger body of the literature. (4) The main focus of the review was studies performed in healthy participants. An inherent assumption is that such studies are focused on healthy kidneys at high altitude. However, the literature suggests that kidney function at high altitude is poor, even in apparently healthy individuals (Hurtado-Arestegui et al., 2017), highlighting the challenges in the study of renal adaptation.

## Future Studies

In the context of limitation 4, the study of renal adaptation to hypoxia in healthy animal models may provide the field with more controlled insights into how a healthy organism adapts to high altitude with potential insights to human adaptation. Indeed, some very interesting studies carried out in the past demonstrate a temporal pattern in renal functional and hemodynamic responses to hypoxic exposure in the laboratory. For example, the immediate response of anesthetized rats to breathing 12% oxygen was a drop in mean arterial blood pressure and an anti-diuresis and anti-natriuresis (Neylon et al., 1995). On the other hand, renal hemodynamic and excretory function was completely normal in animals after 3–5 weeks of exposure to 12% oxygen. These observations were consistent with an increase in hematocrit, which may well have normalized kidney oxygen delivery, kidney oxygen tension and thereby kidney function (Neylon et al., 1997). Unfortunately, these studies do not capture the acute portion of the renal adaptatory response in the 0–3 weeks period. In a more recent study, chronic exposure of rats to hypoxia resulted in hypertension, structural alterations in the renal vasculature (glomerular hypertrophy), macrophage infiltration, interstitial collagen deposition and tubulointerstital injury (Mazzali et al., 2003). Importantly, some of these changes in kidney structure began as early as 6 h into the exposure. Placing this study in the context of the previous studies by Neylon et al. (1995, 1997), tubular interstitial injury and inflammation may present before a deterioration in renal function. Further studies are needed to elucidate these temporal relationships and to establish effects on intrarenal oxygen tension and consumption. It will be important to establish if changes in renal oxygen tension and consumption precede or coincide with structural and functional changes in experimental models exposed to chronic hypoxia.

## Conclusion

The systematic review examined the effect of high altitude on renal physiological function. From the review, we conclude:

1. The physiological response to hypoxia at high altitude evokes a whole body, multi organ system response that acts in concert to maintain tissue oxygen perfusion in an environment where the partial pressure of oxygen is considerably lower.
2. The hormonal response to hypoxia appears dependent upon the magnitude of the hypoxic stimulus and the cumulative duration of exposure. The sustained up-regulation and secretion of erythropoietin seems to be related to the magnitude of the hypoxic stimulus. Erythropoietin expression and synthesis are largely driven by the oxygen tension at the cortico-medullary border. The observations made in studies discussed in this review suggest that the magnitude of the hypoxic stimulus may have detrimental effects of equal magnitude on cortico-medullary oxygen tension and thereby erythropoietin expression. What is presently unclear is whether the hypoxic stimulus at altitude is solely and directly responsible for the reduction in cortico-medullary oxygen tension or whether it can indirectly impact upon renal oxygen tension through effects on renal blood flow and renal oxygen consumption. On the other hand, sustained increases in renin, aldosterone, vasopressin and norepinephrine are related to duration of exposure to moderate altitudes. In light of these data, it is possible that hormones such as Angiotensin II and aldosterone have stimulatory effects on erythropoietin expression because both hormones affect renal tissue oxygen tension (Ang II via effects on renal blood flow and aldosterone via effects on renal oxygen consumption) (Stein et al., 2012; Patinha et al., 2016).
3. Renal hemodynamic control is complex. The initial response may be a compensation for the respiratory system (i.e., hypoxic ventilatory response); however, with time, the hemodynamic response appears to be more related to glomerular filtration rate. Renal plasma flow, renal blood flow, and filtration fraction, which are normally tightly regulated, are markedly changed at high altitude (presumably, in order to maintain glomerular filtration rate).
4. Lastly, limited evidence is available to understand the relationship between the excretory function of the kidney and renal oxygen homeostasis at high altitude. The oxygen cost of sodium reabsorption in the distal nephron is high and relatively inefficient. However, it is unclear whether this is the case at exposure to high altitude. Further evidence related to intrarenal oxygen consumption, the efficiency of sodium transport, and basal metabolism in the kidney at high altitude is needed to fully delineate the issue.

## Data Availability Statement

The original contributions presented in the study are included in the article, further inquiries can be directed to the corresponding author.

## Author Contributions

LP and JO'N contributed the conception, design of the study, independently screened, and selected articles to be included. All authors contributed the data analysis, interpretation, participated in the revision of the manuscript, and have read and approved the submitted version. LP wrote the first draft of the manuscript, with the support of JO'N and K'OH.

## Conflict of Interest

The authors declare that the research was conducted in the absence of any commercial or financial relationships that could be construed as a potential conflict of interest.
